# Extension of the generalized disequilibrium test to polytomous phenotypes and two-locus models

**DOI:** 10.3389/fgene.2014.00258

**Published:** 2014-08-08

**Authors:** Alexandre Bureau, Jordie Croteau, Yvon C. Chagnon, Marc-André Roy, Michel Maziade

**Affiliations:** ^1^Département de Médecine Sociale et Préventive, Université LavalQuébec, QC, Canada; ^2^Centre de Recherche de L'Institut Universitaire en Santé Mentale de QuébecQuébec, QC, Canada; ^3^Département de Psychiatrie et Neurosciences, Université LavalQuébec, QC, Canada

**Keywords:** conditional likelihood, endophenotype, family-based association, kinship, major psychosis, polytomous logistic model, score test

## Abstract

We extend the usual logistic model between a dichotomous phenotype and an allele count in two ways: a polytomous phenotype with *K* > 2 levels, and modeling of allele counts at two unlinked marker loci. Inference is based on within-family information to guard against potential bias due to population genetic structure. Score tests of the model coefficients taking into account the correlation between relatives in entire pedigrees are derived as an extension of the Generalized Disequilibrium Test (GDT). Simulations confirm that the tests have the expected statistical properties, and that their power exceeds that of the GDT under a favorable scenario. The score tests are illustrated with candidate genetic markers, a major psychosis phenotype and a cognitive endophenotype in large kindreds from Eastern Quebec.

## 1. Introduction

Studies of the association between a phenotype and genetic markers are commonly performed on the members of families of various sizes. While methods to estimate association parameters and test the null hypothesis of absence of association (possibly coupled with absence of genetic linkage) with dichotomous phenotypes in family samples are well developed (see for instance chapter 12 of Ziegler and König, 2010), methods are lacking to analyze polytomous phenotypes. Such phenotypes can arise when a disease has multiple subtypes (Guey et al., 2010) or when two dichotomous phenotypes are considered simultaneously. The latter occurs when endophenotypes are measured in genetic studies to better capture phenotypic complexity. Endophenotypes are traits related to a disease and believed to be influenced by fewer genes (Gottesman and Gould, 2003). A dichotomous disease status and a dichotomous endophenotype create a four category phenotype. Comparisons between analyzing a polytomous phenotype vs. a dichotomous one have not been done for family studies due to the lack of analysis methods for polytomous phenotypes.

We focus in this paper on a within-family analysis, conditional on phenotype and genotype observed in each family. Such approach is well known to protect against confounding due to population stratification. Families where multiple phenotypic categories are represented provide the most information on the relationship between a polytomous phenotype and genetic markers. Since families extending over multiple generations typically need to be recruited to obtain a large number of phenotyped subjects, we required that the methods for dichotomous traits that we generalize to polytomous traits be applicable to extended families. For a score test of association, we selected the Generalized disequilibrium test (GDT) of Chen et al. (2009).

In previous work, we showed by simulation that conditioning on a marker at a known disease susceptibility locus increased power to detect linkage to new loci interacting with that disease susceptibility locus (Bureau et al., 2009, 2012). Similar power gains are expected in association analysis, as conditioning on a known environmental risk factor increases power to detect loci interacting with the exposure (Kraft et al., 2007). Models involving genetic markers at two distinct loci are needed for analyses conditional on the genotype of known disease susceptibility markers and also to model the relationship between pairs of loci. Multi-category phenotypes present a larger realm of possibilities of interplay between multiple loci than dichotomous traits, making multilocus modeling even more important to capture the actual effects. This is why we derive score tests under two-locus models, with one marker at each locus, in addition to one-locus models. The Type I error and the power of tests of various combinations of regression coefficients are assessed using simulation. The tests are also illustrated with candidate genetic markers, a major psychosis phenotype and a cognitive endophenotype in the Eastern Quebec kindred study.

## 2. Methods

We extend the GDT of Chen et al. (2009) in two ways: by allowing the outcome *Y* to have *K* > 2 levels, and by allowing the odds of the outcome categories to depend on two or more variables *X*, coding the genotype of markers at two mutually unlinked marker loci. As in the original GDT, *X* represents the count of a particular form of the DNA sequence at the marker, called allele. We begin by deriving the score statistic from the conditional likelihood for a polytomous outcome *Y* with a general vector *X* of allelic count terms (possibly including product terms). Then we derive expressions for particular forms of terms in *X*.

The polytomous model for subject *i* with a general *X_i_* vector can be written

(1) $$\log(\frac{P[Y_{i}=k|X_{i}]}{P[Y_{i}=K|X_{i}]})=\mu_{k}+{\underset{˜}{\beta^{\prime }}}_{k}X_{ki},k=1,\cdots ,K-1$$

where *X_ki_* is the sub-vector of *X_i_* containing the allelic terms related to level (category) *k* and $\underset{˜}{\beta }$*_k_* the sub-vector of the full coefficient vector $\underset{˜}{\beta }$ applicable to level *k* (in this general formulation, β coefficients can either be distinct for each level *k* or can be common to multiple levels of *k*).

Without loss of generality, we assume that the *n* genotyped pedigree members with an observed phenotype are ordered such that the first *n*_1_ subjects are in outcome category *Y* = 1, the *n*_2_ following subjects are in outcome category *Y* = 2 and so on up to the last *n_K_* subjects with *Y = K*.

With *K* = 2 and a single *X* ($\underset{˜}{\beta }$_1_ = β a scalar, without covariates), Chen et al. (2009) showed that the contribution of the family to the score statistic from the conditional likelihood *P* to test the null hypothesis β = 0 has the form:

(2) $$S^{GDT}=\frac{\partial \log P}{\partial \beta }{|}_{\beta =0}=\frac{1}{n}\sum_{i=1}^{n_{1}}\sum_{j=n_{1}+1}^{n}\left(X_{i}-X_{j}\right)$$

We show in Supplementary Material that the contribution of a family to the score statistic for the coefficient β_*h*_ component of $\underset{˜}{\beta }$ when testing the global null hypothesis that the full $\underset{˜}{\beta }$ = 0 under a polytomous model is:

(3) $$\begin{array}{cc} \begin{array}{l} S^{\left(h\right)}=\frac{\partial \log P}{\partial \beta_{h}}|{}_{\underset{˜}{\beta }=0}=\frac{1}{n}[\sum_{i=1}^{n_{1}}\sum_{j=n_{1}+1}^{n}\left(X_{1i}^{\left(h\right)}-X_{1j}^{\left(h\right)}\right)+\cdots \\ \;+\sum_{i=n-(n_{K-1}+n_{K})+1}^{n-n_{K}}\sum_{j\in E_{K-1}}\left(X_{(K-1)i}^{\left(h\right)}-X_{(K-1)j}^{\left(h\right)}\right)] \end{array} & \;\left(3\right) \end{array}$$

where *E*_*K* − 1_ = {1,···,*n* − (*n*_*K* − 1_ − *n_K_*), *n−n_K_* + 1,···,*n*} and *X^(h)^_ai_* is the slice of *X_ai_* related to the coefficient β_h_. If β_h_ is involved only in the logistic function between levels *a* and *K*, then the score statistic simplifies to:

(4) $$S^{\left(h\right)}=\frac{\partial \log P}{\partial \beta_{h}}{|}_{\underset{˜}{\beta }=0}=\frac{1}{n}\sum_{i\in E_{a}}\sum_{j\in E_{a}^{c}}\left(X_{ai}^{\left(h\right)}-X_{aj}^{\left(h\right)}\right)$$

where *E_a_* = {*n_1_* + ··· + *n*_*a*−1_ + 1, ···, *n_1_*+ ··· + *n_a_*} for *a* > 1 and *E*_1_ = {1 ··· *n*_1_}.

The advantage of expression 3 is that a closed-form expression for the variance of *S^(h)^* and the covariance of *S^(g)^* and *S^(h)^* for coefficients β*_g_* and β_*h*_ can be derived, following the steps of Chen et al. It is also easier to interpret. When the tested coefficient belongs to the logistic function attached to a single outcome category and the score statistic reduces to expression 4, it is a contrast of the value of the corresponding *X* term between subjects in the outcome category and subjects in all other categories.

Letting *v[S]* be an estimate of the variance-covariance matrix of S, the null hypothesis that $\underset{˜}{\beta }$ = 0 can then be tested with the statistic

$$T=S^{\prime }v{\left[S\right]}^{-1}S$$

which follows a χ^2^ distribution with degrees of freedom equal to the rank of $\underset{˜}{\beta }$ under the null.

When testing the sub null hypothesis β_*h*1_ = ··· = β_*h_m_*_ = 0 for any subset of indices *h*_1_, ···, *h_m_*, the other coefficients are free to differ from 0 and the derivation in Supplementary Material no longer applies. We adopt here the approach Chen et al. (2009) apply to model covariates, which is to weight the pairwise differences according to a model of the outcome *Y* as a function of the predictors with free coefficients under the null hypothesis. The score statistic for the component β_*h*_ of the subset of coefficients tested then becomes

(5) $$S^{\left(h\right)}=\frac{\partial \log P}{\partial \beta_{h}}{|}_{\beta_{h_{1}}=\cdots =\beta_{h_{m}}=0}=\sum_{i\in E_{a}}\sum_{j\in E_{a}^{c}}C_{ij}\left(X_{ai}^{\left(h\right)}-X_{aj}^{\left(h\right)}\right)$$

where the weights *C_ij_* can be derived from score equations for β_*h*_ under the pairwise formulation of Liang and Stewart (1987) (see Supplementary Material), leading to the following functions of the coefficients $\underset{˜}{\alpha }$ of a polytomous logistic model of *Y* as a function of the predictors *X^(c)^*, *c* = {*l : l* ∉ (*h*_1_,···,*h_m_*)} when the variability from estimating the $\underset{˜}{\alpha }$ is neglected:

(6) $$C_{ij}=\frac{2}{N}\frac{1}{\left(1+exp\left\{{\left(X_{i}^{\left(c\right)}-X_{j}^{\left(c\right)}\right)}^{\prime }\left({\underset{˜}{\alpha }}_{Y_{i}}-\;{\underset{˜}{\alpha }}_{Y_{j}}\right)\right\}\right)}$$

where $\underset{˜}{\alpha }$*_K_* = 0.

Adapting Chen et al. (2009)'s Equation 2 from the dichotomous to the polytomous case gives the following expression for the weights instead:

(7) $$C_{ij}=\frac{8}{N}\frac{exp\left\{{\left(X_{i}^{\left(c\right)}-X_{j}^{\left(c\right)}\right)}^{\prime }\left(\;{\underset{˜}{\alpha }}_{Y_{i}}-\;{\underset{˜}{\alpha }}_{Y_{j}}\right)\right\}}{{\left(1+exp\left\{{\left(X_{i}^{\left(c\right)}-X_{j}^{\left(c\right)}\right)}^{\prime }\left({\underset{˜}{\alpha }}_{Y_{i}}-\;{\underset{˜}{\alpha }}_{Y_{j}}\right)\right\}\right)}^{3}}$$

We estimate the coefficients $\underset{˜}{\alpha }$ using generalized estimating equations (GEEs) with an independence working correlation matrix. With this approach the null hypothesis that the component β_*h*_ = 0 can be tested with the statistic

$$Z^{\left(h\right)}=S^{\left(h\right)}/\sqrt{v[S^{\left(h\right)}]}$$

which follows approximately a standard normal distribution under the null, when the weights are defined in such a way that the expectation of *S^(h)^* is 0. The weight definition will only have an impact on power. The joint null hypothesis β_*h*_1__ = ··· = β*_h_m__* = 0 for any subset of indices *h*_1_, ···, *h_m_* can be tested with the statistic

(8) $$T=\left(S_{h_{1}},\cdots ,S_{h_{m}}\right){\left(v[S_{h_{1}},\cdots ,S_{h_{m}}]\right)}^{-1}{\left(S_{h_{1}},\cdots ,S_{h_{m}}\right)}^{\prime }$$

which follows approximately a χ^2^ distribution with *m* degrees of freedom under the null.

The variance of *S^(h)^* depends on whether the null hypothesis refers only to absence of association, or to absence of genetic linkage and association. In the first case, the null distribution of *S^(h)^* allows genetic linkage at the locus, and the identical-by-descent (IBD) sharing proportions in the variance estimate must be the actual IBD sharing proportions at the locus π*_hij_* (Chen et al., 2009). For the second case, or when IBD is unknown, π*_hij_* can be substituted by twice the kinship coefficients ϕ_*ij*_, which is constant at all loci. The general expression for the variance of *S^(h)^* and covariance between *S^(h)^* and *S^(g)^* is given in Supplementary Material. When *S^(h)^* takes the form 4, *X^(h)^_ai_* is a main effect term, say *X*_1_, and the actual IBD sharing proportions π*_hij_* are used then

(9) $$\begin{array}{l} Var[S^{\left(h\right)}]=Var\left[\sum_{i\in E}\sum_{j\in E^{c}}C_{ij}(X_{ai}^{\left(h\right)}-X_{aj}^{\left(h\right)})\right]\\ \;=\sum_{i,k\in E}\sum_{j,l\in E^{c}}C_{ij}C_{kl}Cov[X_{ai}^{\left(h\right)}-X_{aj}^{\left(h\right)},X_{ak}^{\left(h\right)}-X_{al}^{\left(h\right)}]\\ \;=\sum_{i,k\in E}\sum_{j,l\in E^{c}}C_{ij}C_{kl}(Cov[X_{1i},X_{1k}]+Cov[X_{1j},X_{1l}]\\ \;-\;Cov[X_{1i},X_{1l}]-Cov[X_{1j},X_{1k}])\\ \;=\sum_{i,k\in E}\sum_{j,l\in E^{c}}C_{ij}C_{kl}\left(\pi_{1ik}+\pi_{1jl}-\pi_{1il}-\pi_{1jk}\right)\sigma_{1}^{2} \end{array}$$

The within-family variance of *X*_1_, σ^2^_1_, is estimated as described in Supplementary Material to obtain the estimate *v[S^(h)^]* of *Var[S^(h)^]*. With equal weights for all pairs, the computation involving the IBD sharing probabilities can be simplified as explained in Supplementary Material.

When *X^(h)^_ai_* is instead a product term, say *X*_1_*X_2_*, then

$$\begin{array}{l} Var[S^{\left(h\right)}]=Var\left[\frac{1}{n}\sum_{i\in E}\sum_{j\in E^{c}}\left(X_{ai}^{\left(h\right)}-X_{aj}^{\left(h\right)}\right)\right]\\ \;=\frac{1}{n^{2}}\sum_{i\in E}\sum_{j\in E^{c}}\sum_{k\in E}\sum_{l\in E^{c}}\\ \;\left(\begin{array}{l} Cov[X_{1i}X_{2i},X_{1k}X_{2k}]+Cov[X_{1j}X_{2j},X_{1l}X_{2l}]\\ -Cov[X_{1i}X_{2i},X_{1l}X_{2l}]-Cov[X_{1j}X_{2j},X_{1k}X_{2k}] \end{array}\right)\\ \;=\frac{1}{n^{2}}\sum_{i\in E}\sum_{j\in E^{c}}\sum_{k\in E}\sum_{l\in E^{c}}\\ \;\left(\pi_{1ik}\pi_{2ik}+\pi_{1jl}\pi_{2jl}-\pi_{1il}\pi_{2il}-\pi_{1jk}\pi_{2jk}\right)\sigma_{12}^{2} \end{array}$$

where the within-family variance of the product term *X*_1_ *X*_2_, σ^2^_12_, is estimated as described in Supplementary Material.

### 2.1. Application to the joint modeling of two dichotomous traits using two-locus models

The joint analysis of two dichotomous traits represents an important special case of a polytomous phenotype with four categories. We illustrate such a phenotype by referring to a dichotomous disease trait *Y*_2_ and a dichotomous endophenotype *Y*_1_, as defined in the introduction.

We consider here polytomous models for two markers at unlinked loci which may interact to cause the disease and endophenotype impairment. We assume that association of locus 1 to the endophenotype impairment *Y*_1_ = 1 and possibly to the disease *Y*_2_ = 1 has already been established, and that we want to detect locus 2, which is undetectable in single-locus analyses, by conditioning on locus 1 with which it interacts. This leads to null hypotheses on a subset of coefficients tested with a statistic as defined in Equation 8.

A first option is to use the full model with distinct coefficients for each disease/endophenotype combination contrasted to the reference category of absence of both the disease and endophenotype impairment. This model is:

(10) $$\begin{array}{l} \log\left(\frac{P[Y_{1}=1,Y_{2}=0|X_{1},X_{2}]}{P[Y_{1}=0,Y_{2}=0|X_{1},X_{2}]}\right)\\ \;=\beta_{10}+\beta_{11}X_{1}+\beta_{12}X_{2}+\beta_{13}X_{1}X_{2}\\ \log\left(\frac{P[Y_{1}=0,Y_{2}=1|X_{1},X_{2}]}{P[Y_{1}=0,Y_{2}=0|X_{1},X_{2}]}\right)\\ \;=\beta_{20}+\beta_{21}X_{1}+\beta_{22}X_{2}+\beta_{23}X_{1}X_{2}\\ \log\left(\frac{P[Y_{1}=1,Y_{2}=1|X_{1},X_{2}]}{P[Y_{1}=0,Y_{2}=0|X_{1},X_{2}]}\right)\\ \;=\beta_{30}+\beta_{31}X_{1}+\beta_{32}X_{2}+\beta_{33}X_{1}X_{2} \end{array}$$

The null hypothesis of the conditional test of locus 2 given locus 1 under the full model is formulated as:

(11) $$\beta_{12}=\beta_{13}=\beta_{22}=\beta_{23}=\beta_{32}=\beta_{33}=0$$

When the null is rejected, insights on the phenotype category driving the signal can be obtained by examining the *Z* statistics for each coefficient and the *p*-values associated to the tests of the subsets of coefficients (β_12_, β_13_),(β_22_, β_23_) and (β_32_, β_33_).

One can also postulate a model for a particular form of interaction between the two loci. We consider a model which we call the endophenotype-to-disease model where an allele at locus 1 increases susceptibility to the endophenotype impairment *Y*_1_ = 1 and possibly to the disease *Y*_2_ = 1, and an allele at locus 2 increases susceptibility to the disease in carriers of gene 1 susceptibility genotypes (at higher risk of the endophenotype impairment). For that model we express allele counts as proportion of a given allele in a genotype, taking values 0, $\frac{1}{2}$ and 1. The model is then written as:

(12) $$\begin{array}{l} \log\left(\frac{P[Y_{1}=1,Y_{2}=0|X_{1},X_{2}]}{P[Y_{1}=0,Y_{2}=0|X_{1},X_{2}]}\right)\\ \;=\beta_{10}+\beta_{11}X_{1}+\beta_{e}X_{1}(1-X_{2})\\ \log\left(\frac{P[Y_{1}=0,Y_{2}=1|X_{1},X_{2}]}{P[Y_{1}=0,Y_{2}=0|X_{1},X_{2}]}\right)\\ \;=\beta_{20}+\beta_{21}X_{1}\\ \log\left(\frac{P[Y_{1}=1,Y_{2}=1|X_{1},X_{2}]}{P[Y_{1}=0,Y_{2}=0|X_{1},X_{2}]}\right)\\ \;=\beta_{30}+\beta_{31}X_{1}+\beta_{33}X_{1}X_{2} \end{array}$$

We keep the same notation for the coefficients as in the full model, except for the coefficient β_*e*_, which represents the effect on the risk of the endophenotype impairment in non-carriers of the locus 2 tested allele. When the endophenotype-to-disease model holds, the coefficients β_33_ and β_*e*_ are of the same sign. The marginal association of *X*_2_ to the endophenotype impairment under that model will typically be small. Its direction and magnitude depend on the values of β_33_ and β_*e*_ and the distribution of *X*_1_.

The null hypothesis of the conditional test of locus 2 given locus 1 under the above model is formulated as:

(13) $$\beta_{e}=\beta_{33}=0$$

The alternative hypothesis can be restricted to

$$\beta_{e}>0,\beta_{33}>0\cup \beta_{e}<0,\beta_{33}<0$$

or a general alternative can be considered, but the alternative space then contains models outside of the conceptual model formulated above.

Alternatively, detection of locus 2 can be attempted by testing a single interaction parameter between *X*_1_ and *X*_2_, as in the context of a genetic analysis conditional on an environmental exposure (Kraft et al., 2007). Here the interaction parameter for the logistic function contrasting the disease and endophenotype impairment category to the reference category β_33_ is the most promising to test to detect effects on the disease and endophenotype impairment jointly.

### 2.2. Software implementation

We have implemented the extension of the GDT to polytomous phenotypes and two loci in the R package fat2Lpoly, standing for Family-based Association Test for 2 Loci and Polytomous phenotypes available on the CRAN archive at CRAN.R-project.org/package = fat2Lpoly. A function is provided to read phenotype and genotype data, variable names and IBD sharing proportions (if applicable) from input files in the Merlin/QTDT format (www.sph.umich.edu/csg/abecasis/Merlin/tour/input_files.html) and convert them into R objects. Alternatively, R objects made by the user in the same format can be provided as input. Functions are provided to setup design matrices for the full two-locus polytomous model, the one-locus polytomous model and the disease-to-endophenotype model. User-defined functions setting-up customized design matrices can be provided instead of these pre-defined functions.

### 2.3. Evaluation by simulation of the proposed hypothesis tests under two-locus models

**Figure 1:**
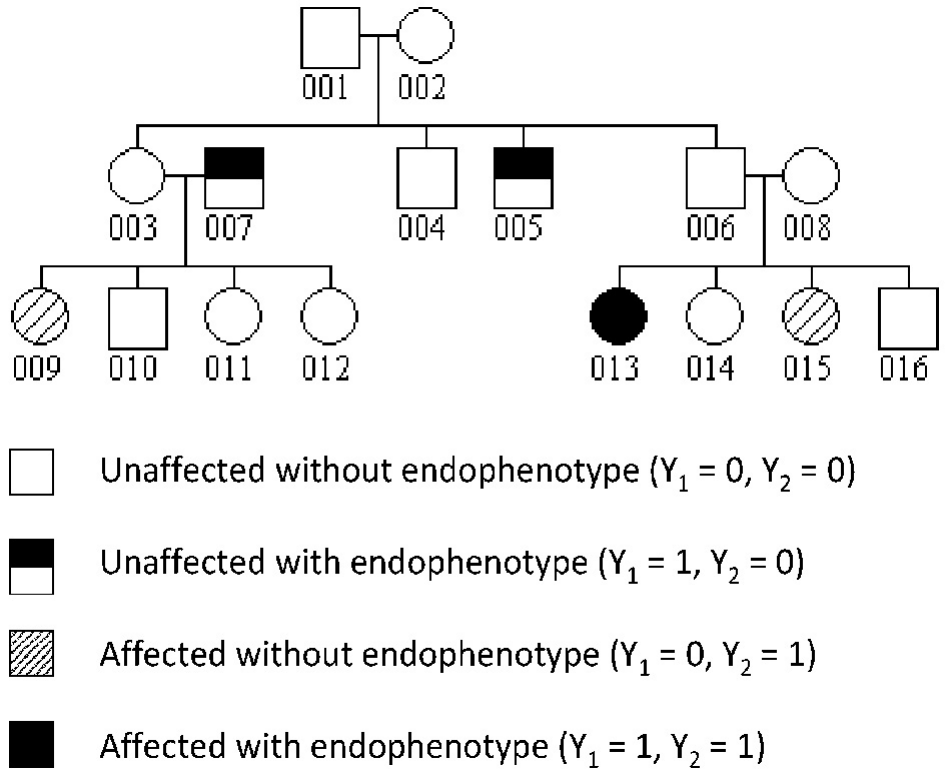
**Structure of simulated families with an example of phenotype realization**.

The family structure used in the simulations is a 3-generation 16-member family depicted in Figure 1. The disease and endophenotype status of all family members was assumed to be observed. We generated genotype data for genetic variants with two alleles such as single nucleotide polymorphisms (SNPs) at two independent loci. The genotypes of pedigree founders were sampled under Hardy-Weinberg equilibrium using risk allele frequencies (RAFs) of 0.1 at locus 1 and 0.3 at locus 2. The transmission of alleles to their descendants was then simulated following the rules of Mendelian inheritance. Two dichotomous phenotypes *Y*_1_ and *Y*_2_ were generated in a two-step approach: we first simulated from the distribution of *Y*_*i*1_ for each subject *i* by summing over *Y*_*i*2_ in a polytomous model, then from the distribution of the vector *Y*_2_|*Y*_1_. In the model to simulate *Y*_*i*2_|*Y*_1_, *Y*_1_ is treated as a vector of fixed effect, with the effect of the endophenotype of subject *h*, *Y*_*h*1_, modulated by the kinship coefficient ϕ_*ih*_ between *i* and *h*. An additive polygenic effect on the logit of *Y*_2_ was also included. The model can be written:

(14) $$\begin{array}{l} \log\left(\frac{P[Y_{i2}=1|Y_{1},X,U]}{P[Y_{i2}=0|Y_{1},X,U]}\right)=\gamma^{\prime }(X_{i},Y_{i1})+U_{i}\\ \;+\;\alpha \sum_{h\neq i}^{n}\left(Y_{h1}-\nu \right)\phi_{ih} \end{array}$$

(15) $$U\sim N(0,\sigma^{2}\Phi )$$

where γ′(*X_i_*, *Y*_*i*1_) in an abbreviated expression of the model for the disease phenotype given the genotype at major loci and endophenotype status of subject *i* derived from a polytomous model and Φ is the kinship matrix between the family members. The parameter σ^2^ controls the degree of polygenic dependence between the disease status *Y*_2_ of the family members and the parameter α the degree of genetic dependance of *Y*_2_ on *Y*_1_ not captured by the genotype at the loci in the model. The parameter ν, between 0 and 1, determines the relative importance of the risk increase 1 − ν due to observing an endophenotype impairment and the risk decrease −ν due to observing the normal level of the endophenotype in a relative. We note this simulation scheme is meant to reproduce the association between disease phenotype and endophenotype status of relatives, not to represent a causal mechanism. Among the simulated families, we kept those with at least a cousin pair with *Y*_2_ = 1, i.e., affected by the disease to mimic the ascertainment process of families in a genetic study.

We simulated two scenarios of population origin of the sample: (1) homogeneity: the sample came from a single population where the phenotypes were generated under the polytomous model presented in Table 1. Under this models and with the above RAFs, the disease had a population prevalence of 0.0076 and the endophenotype impairment a prevalence of 0.128; (2) heterogeneity: the sample was a mixture of families from two populations, both represented in equal proportions. In population 1, all intercept coefficients in Table 1 were reduced by 0.5, while in population 2 they were increased by 0.5. This resulted in disease prevalences of 0.005 in population 1 and 0.012 in population 2, and endophenotype impairment prevalences of 0.082 in population 1 and 0.194 in population 2.

**Table 1:** **Regression coefficients of the example polytomous model**.

**Coef**.	**Value**	**Coef**.	**Value**	**Coef**.	**Value**	**Coef**.	**Value**
β_10_	−2	β_11_	log(2)	β_12_	0	β_13_	−log(2)
β_20_	−5.5	β_21_	0	β_22_	0	β_23_	0
β_30_	−5.5	β_31_	0	β_32_	0	β_33_	log(16)

To verify the Type I error of tests of association to locus 2 under the null hypothesis of no association to locus 2, but in presence of genetic linkage at that locus, we generated an additional biallelic variant at locus 2 independent from the causal variant at that locus, i.e., in linkage equilibrium with it. In the homogeneous population, the minor allele frequency of that marker was equal to the RAF of the causal variant, but in the mixture of two populations the minor allele frequency was 0.1 in population 1 and 0.5 in population 2, creating population structure at that locus. For the power evaluation, we tested association to the actual causal variant at locus 2.

The tests evaluated include the tests of the null hypotheses 11 which we denote “cpoly,” 13 which we denote “(β_*e*_, β_33_),” and β_33_ = 0. We also evaluated a single locus polytomous model (model 11 with *X*_2_ only). The coefficients in that model are labeled $\underset{˜}{\beta }$, and we tested the null hypotheses $\underset{˜}{\beta }$ = 0 as well as β_3_(1*L*) = 0. For the evaluation of the Type I error, Wald tests of the coefficients of the one locus model based on GEEs were also performed. However, these tests were not used for the power comparison, since they had inflated Type I error under our heterogeneity scenario where population stratification was present.

In presence of population stratification, previously available valid tests are restricted to a dichotomous outcome and a single marker. Analysis options are then limited to testing association of a single marker to the dichotomous endophenotype *Y*_1_ and disease status *Y*_2_, either in the full sample or, in the case of *Y*_2_, in a stratum defined by *Y*_1_. This is akin to the strategy for detecting modifier genes conferring susceptibility to a specific phenotype (i.e., the disease) consisting in testing association to the specific phenotype among subjects with a broader phenotype (i.e., the endophenotype impairment) (Bureau et al., 2012). We therefore compared the power of various tests derived under our extension of the GDT against the single marker GDT for dichotomous outcomes applied to the locus 2 causal variant with three phenotype definitions: (1) the disease status *Y*_2_ (standard analysis noted simply GDT), (2) the disease status *Y*_2_ in the subset of subjects with *Y*_1_ = 1 (endophenotype impairment), setting the phenotype of other subjects to unknown (GDTc), and (3) the endophenotype status *Y*_1_ (GDTe). We also compared our tests to score tests of coefficients of the usual two-locus logistic model for a dichotomous trait:

(16) $$\log\left(\frac{P[Y=1|X_{1},X_{2}]}{P[Y=0|X_{1},X_{2}]}\right)=\eta_{0}+\eta_{1}X_{1}+\eta_{2}X_{2}+\eta_{3}X_{1}X_{2}$$

The 2 d.f. test of the null hypothesis η_2_ = η_3_ = 0 is denoted “cdisease” when the phenotype tested is *Y*_2_ and “cendo” when the phenotype tested is *Y*_1_.

## 3. Results

### 3.1. Evaluation of the type I error

The Type I error was evaluated on 1000 replicate samples of 100 families. The results of the simulation under the null hypothesis in Table 2 show that the nominal Type I error rate was respected under both scenarios for all test statistics from our polytomous extension of the GDT. The Type I error rates of the tests conditional on locus 1 were similar for weight definitions 6 and 7, so only results for the former are shown. They were both below the nominal level, making these tests conservative. By contrast, the Type I error of the Wald tests based on GEE estimates were at nominal level only under the homogeneous sample scenario, and were severely inflated under the heterogeneous sample scenario.

**Table 2:** **Estimations of Type I error on 1000 replicate samples of 100 families**.

	**GEE**		**Conditional likelihood**				
	**Single locus**		**Single locus**		**Given other locus^a^**		
	**β_3_(1*L*)**	**$\underset{˜}{\beta }$(1*L*)**	**β_3_(1*L*)**	**$\underset{˜}{\beta }$(1*L*)**	**β_33_**	**(β_*e*_,β_33_)**	**cpoly**
**HOMOGENEOUS POPULATION**							
α = 0.01:	0.009	0.015	0.006	0.012	0.001	0.001	0.007
α = 0.05:	0.053	0.060	0.051	0.045	0.019	0.003	0.029
**MIXTURE OF TWO POPULATIONS**							
α = 0.01:	0.102	0.762	0.012	0.010	0.002	0.001	0.007
α = 0.05:	0.237	0.906	0.048	0.053	0.025	0.003	0.033

a *subject pairs were weighted using expression 6*.

### 3.2. Evaluation of the power

**Figure 2:**
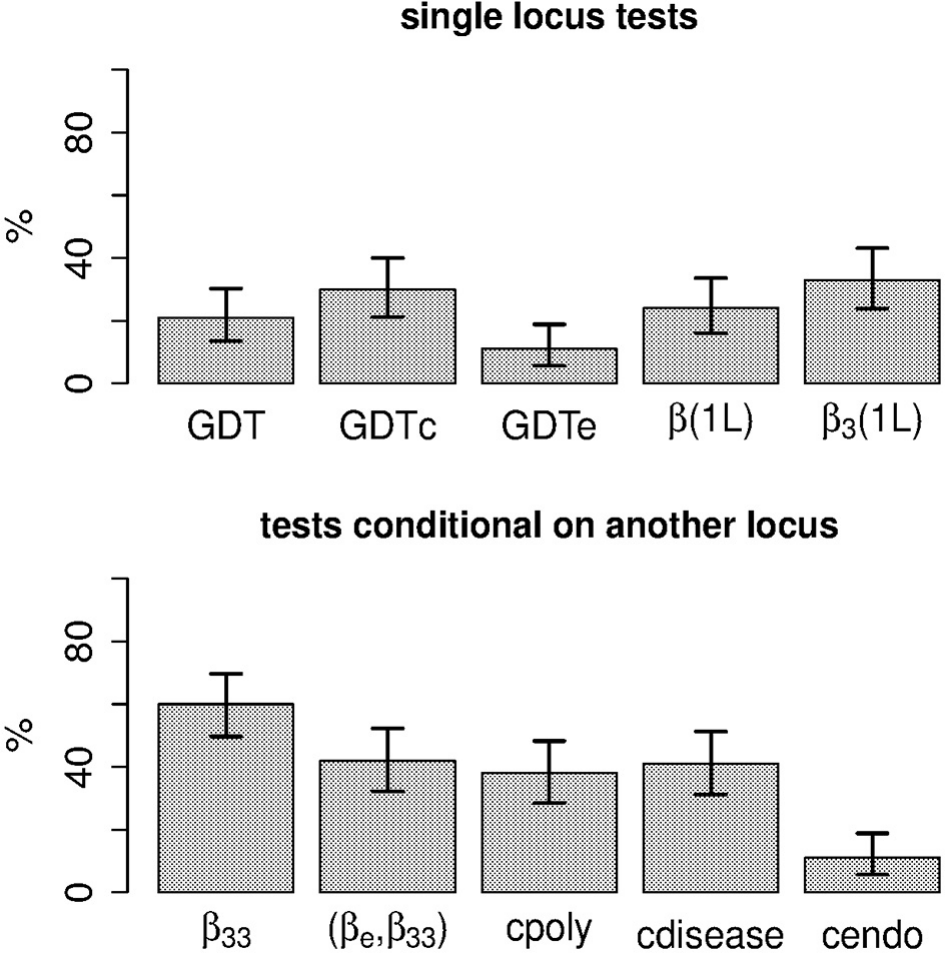
**Power of various within-family score tests to detect locus 2**. See text for definitions of the acronyms of the tests. For tests conditional on another locus, subject pairs were weighted using expression 6.

Under the simulated scenario the endophenotype-to-disease model holds. While the test of the null hypothesis 13 has some power, testing β_33_ = 0 (the interaction parameter for the combination of disease and endophenotype impairment) achieves the highest power among the tests considered (Figure 2). Using weight definition 7 instead of 6 led to nearly identical power (results not shown). Under this scenario, testing association for the same phenotypic category of the allele count at locus 2 β_3_(1*L*) = 0 or the entire vector $\underset{˜}{\beta }$ = 0 does not provide a measurable power improvement over the GDT applied to the disease status in the subset of subjects with endophenotype impairment. Further comparisons of testing strategies under a variety of scenarios will be reported elsewhere.

### 3.3. Application to major psychosis and visual episodic memory

Schizophrenia (SZ) and bipolar disorder (BP) are two forms of the spectrum of major psychosis (MP), which also includes schizo-affective disorder. SZ and BP co-aggregate in families (Van Snellenberg and de Candia, 2009), and share genetic liability (Cross-Disorder Group of the Psychiatric Genomics Consortium, 2013). Various cognitive domains are widely recognized as endophenotypes of MP (Bora et al., 2009; Ivleva et al., 2010). In the Eastern Quebec kindred study, visual episodic memory (VisEM) was found to be impaired in both SZ and BP patients and non-affected adult relatives of these patients (Maziade et al., 2011). In that same family sample, we recently replicated an association between the T allele of SNP rs1156026 and SZ that we had previously detected in another sample (Bureau et al., 2013). All the elements required for the application of our extension of the GDT to markers genotyped in the family sample are present: a diagnosis within the spectrum of MP as the disease phenotype, a VisEM mesurement dichotomized as presence/absence of deficit as the endophenotype and the SNP rs1156026 as the established risk locus. Given the small number of subjects with cognitive measurements, this analysis is not sufficiently powered to draw conclusions and must be considered illustrative. The small sample size also limited us to an analysis of MP globally, without separating SZ and BP.

VisEM was measured by the performance on the delayed recall of the Rey figure task (Meyers and Meyers, 1995) defining the affected status as being the 4th percentile of the distribution of age and gender matched controls. We retained the 14 informative families defined as containing at least one MP affected subject with a visual memory measurement and subjects in at least one other phenotypic category. Table 3 presents the joint distribution of MP and VisEM in the 133 genotyped subjects from these families along with the frequency of the rs1156026 T allele. Although the frequency of the T allele is greatly increased in subjects with MP and the VisEM impairment compared to normal subjects (and this increase is statistically significant in a population-level comparison) the within-family score test of the corresponding coefficient has a high *p*-value, suggesting that the difference in T allele frequency is mostly between families and not so much within families.

**Table 3:** **Joint distribution of major psychosis and visual episodic memory deficits along with the frequency of the rs1156026 T allele**.

		**VisEM <= 4th perc**				**VisEM > 4th perc**				**Total**	
		***n*_1_**	**Freq T**	***p_GEE_*^a^**	***p_1L_*^b^**	***n*_0_**	**Freq T**	***p_GEE_*^a^**	***p_1L_*^b^**	***n*_._**	**Freq T**
MP	Yes	21 (41%)	0.52	0.0011	0.34	30	0.40	0.040	0.310	51 (38%)	0.45
	No	13 (16%)	0.31	0.97	0.72	69	0.30			82 (62%)	0.30
	Total	34 (26%)	0.44			99	0.33			133	0.36

a *p-values of Wald tests of the coefficients of the one locus polytomous model estimated using generalized estimating equations (GEE)*.

b *p-values of within-family score tests of the coefficients of the one locus polytomous model*.

We tested association to 80 SNPs in genomic regions where genetic linkage to SZ, BP, or MP was previously detected in that family sample on the p arm of chromosomes 6, 8, and 16 and the q arm of chromosomes 12 and 18 (Maziade et al., 2005). We applied the same tests as in the simulation study. SNPs where a *p*-value < 0.05 was obtained in at least one analysis are shown in Table 4.

**Table 4:** **Results for SNPs where a *p*-value < 0.05 was obtained in at least one analysis^a^**.

**SNP**	**Chr**	**Pos (Mb)**		**MAF (*n*)**			
				***Y*_1_ = 0, *Y*_2_ = 0**	***Y*_1_ = 0, *Y*_2_ = **1****	***Y*_1_ = 1, *Y*_2_ = 0**	***Y*_1_ = 1, *Y*_2_ = 1**
rs1087266	6	24.4		0.39 (42)	0.26 (25)	0.60 (5)	0.55 (19)
rs7500550	16	19.1		0.11 (41)	0.16 (25)	0.17 (6)	0.03 (18)
**TESTS *p*-VALUES**							
**SNP**	**GDT**	**GDTc**	**GDTe**	**$\underset{˜}{\beta }$(1*L*)**	**β_33_**	**(β_e_,β_33_)**	**cpoly**
rs1087266	0.48	0.25	0.005	0.005	0.032	0.085	0.006
rs7500550	0.52	0.17	0.57	0.040	0.019	0.064	0.015

a *For tests conditioning on rs1156026 genotypes, subject pairs were weighted using expression 6*.

The results for rs7500550 illustrate that tests of the joint MP-VisEM phenotype conditional on the rs1156026 T allele count can detect associations to SNPs where the test of the MP or VisEM phenotype alone did not. In this case, the rare allele was negatively associated to MP with VisEM impairment with *Z* statistics of −2.66 for the *X*_2_ and −2.34 for the *X*_1_*X*_2_ terms (*p* = 0.0019 for the test of the coefficients of both terms) while it was positively associated to a lesser extent to MP without VisEM impairment with *Z* statistics of 2.54 for the *X*_2_ and 2.07 for the *X*_1_*X*_2_ terms (*p* = 0.005 for the test of the coefficients of both terms). The signal was thus driven by opposite associations to these two phenotypic categories. The signal at rs1087266 was detected by single locus tests with lower *p*-values than by tests conditioning on rs1156026. In that case, testing association with VisEM status was the key to detect the signal. Nonetheless, the conditional test of the polytomous phenotype provides a *p*-value similar to the standard GDT. Given the limited power of the analysis and the number of SNPs tested, these results cannot be considered statistically significant once multiple testing is taken into account.

## 4. Discussion

We have extended the GDT, a score test of genetic association applicable with extended families, to enable testing association with a polytomous phenotype. Another extension is the use of a model of association with two genetic loci, allowing to test association at a locus conditional on the genotype of a marker at a known risk locus, to exploit interaction between the two. A software implementation in the form of a R package has been made freely available. The within-family analysis framework that we adopted has the advantage of protecting against Type I error inflation due to population stratification. Polytomous phenotypes can be more informative than dichotomous ones to detect genetic associations, as illustrated in our simulation study.

The proposed score tests also suffer from limitations. First, score tests provide no estimates of the regression parameters being tested. Conditional maximum likelihood estimation would be applicable only with exchangeable relatives, which is not required for the GDT as explained in Supplementary Material. We are exploring the robustness and power of conditional maximum likelihood estimation in sibships from extended families.

Second, within-family analysis tends to be less powerful than population-level analysis which also exploits between family information. Furthermore, the Type I error of score tests for one locus conditionning on another tends to be conservative even with the weight definition 6 neglecting variability from estimating the $\underset{˜}{\alpha }$. Our simulation studies illustrate that power remains limited despite large sample sizes (1600 subjects in 100 families) and large effect sizes (interaction odds ratios of 16). Extracting the most power from the data is particularly important when phenotypic measures are expensive to obtain, such as the cognitive measurements in our example. Population analyses are then attractive, with an adjustment for population structure using genomewide SNP genotypes (Price et al., 2006). Methods for population analysis of polytomous phenotypes are not well developed, and will be the object of future work.

## Author contributions

Alexandre Bureau defined the research questions, derived the proposed statistical test, wrote part of the R implementation, conceived the simulation study, oversaw the analysis of the major psychosis data and drafted the manuscript. Jordie Croteau wrote part of the R implementation, performed the simulation study and the analysis of the major psychosis data, and created figures and tables. Yvon C. Chagnon oversaw the genotyping of the Eastern Quebec kindred study and contributed to the design of the genetic aspects of that study. Marc-André Roy contributed to the design of the genetic and clinical aspects of the Eastern Quebec kindred study, established diagnosis of patients and made substantial revisions to the manuscript. Michel Maziade designed the genetic and clinical aspects of the Eastern Quebec kindred study, oversaw clinical data collection and established diagnosis of patients. All authors approved the version submitted for publication and agree to be accountable for all aspects of the work.

### Conflict of interest statement

The authors declare that the research was conducted in the absence of any commercial or financial relationships that could be construed as a potential conflict of interest.
