# Supplementary material for: Extension of the generalized disequilibrium test to polytomous phenotypes and two-locus models
Source: Front Genet. 2014 Aug 8;5:258. doi: 10.3389/fgene.2014.00258 (PMC4126369; doi:10.3389/fgene.2014.00258)
Supplement: Supplementary file 1 [file DataSheet1.PDF]

Frontiers in Genetics

Methods Article
28 July 2014

Supplementary Material: Extension of the Generalized Disequilibrium Test to polytomous phenotypes and two-locus models

Alexandre Bureau [1,2,*], Jordie Croteau [2], Yvon C. Chagnon [2], Marc-André Roy [2,3] and Michel Maziade [2,3]

[1] *Département de médecine sociale et préventive, Université Laval, Québec, QC, Canada*
[2] *Centre de recherche de l'Institut universitaire en santé mentale de Québec, Québec, Qc, Canada*
[3] *Département de psychiatrie et neurosciences, Université Laval, Québec, QC, Canada*

Correspondence*:
Alexandre Bureau
Département de médecine sociale et préventive, Université Laval, 1050 rue de la Médecine, local 2457, Québec, QC G1V 0A6 (Canada), alexandre.bureau@fmed.ulaval.ca

1 SUPPLEMENTARY DATA

1.1 DERIVATION OF THE SCORE STATISTIC FOR A POLYTOMOUS OUTCOME WITH K LEVELS

Let Y be the outcome with K levels, where K is the reference level. For $a = 1, \ldots, K$, let N_a be the number of subjects whose level (category) is a. The contribution to the conditional likelihood of one family is

$$
\begin{aligned}
P &= P\left[\sum_{i=1}^{n_1} I(Y_i = 1) = n_1, \ldots, \sum_{i=n-(n_{K-1}+n_K)+1}^{n-n_K} I(Y_i = K-1) = n_{K-1} | N_1 = n_1, \ldots, N_K = n_K\right] \\
&= \frac{P\left[\sum_{i=1}^{n_1} I(Y_i = 1) = n_1, \ldots, \sum_{i=n-(n_{K-1}+n_K)+1}^{n-n_K} I(Y_i = K-1) = n_{K-1}\right]}{\sum_{\mathcal{S}} P\left[\sum_{i \in S_1} I(Y_i = 1) = n_1, \ldots, \sum_{i=n-(n_{K-1}+n_K)+1}^{n-n_K} I(Y_i = K-1) = n_{K-1}\right]} \\
&= \frac{\exp\left\{\sum_{i=1}^{n_1} X_{1i}\underset{\sim}{\beta} + \ldots + \sum_{i=n-(n_{K-1}+n_K)+1}^{n-n_K} X_{(K-1)i}\underset{\sim}{\beta}\right\}}{\sum_{\mathcal{S}} \exp\left\{\sum_{i \in \mathcal{S}_1} X_{1i}\underset{\sim}{\beta} + \ldots + \sum_{i \in \mathcal{S}_{K-1}} X_{(K-1)i}\underset{\sim}{\beta}\right\}}
\end{aligned}
$$

where X_a is the matrix of allelic terms for level (category) a, $\mathcal{S}$ is the set of indices of all the family members under a permutations of these family members, and $\mathcal{S}_a$ is the subset of indices of the subjects in category a in that permutation.

$$\frac{\partial \log P}{\partial \beta_h} = \sum_{i=1}^{n_1} X_{1i}^{(h)} + \ldots + \sum_{i=n-(n_{K-1}+n_K)+1}^{n-n_K} X_{(K-1)i}^{(h)}$$

$$- \quad \frac{\sum_{\mathcal{S}} \left(\sum_{i \in \mathcal{S}_1} X_{1i}^{(h)} + \ldots + \sum_{i \in \mathcal{S}_{K-1}} X_{(K-1)i}^{(h)} \right) \exp\left\{ \sum_{i \in \mathcal{S}_1} X_{1i} \underset{\sim}{\beta} + \ldots + \sum_{i \in \mathcal{S}_{K-1}} X_{(K-1)i} \underset{\sim}{\beta} \right\}}{\sum_{\mathcal{S}} \exp\left\{ \sum_{i \in \mathcal{S}_1} X_{1i} \underset{\sim}{\beta} + \ldots + \sum_{i \in \mathcal{S}_{K-1}} X_{(K-1)i} \underset{\sim}{\beta} \right\}}$$

where $X_{ai}^{(h)}$ is the slice of X_{ai} related to β_h. This likelihood implies that the family members are exchangeable, which is a reasonnable assumption only when their relationships to each other are all the same, e.g. they are all siblings or all first cousins. When this is not the case, the likelihood is misspecified, but the score statistics remains unbiased under the null, and the varying degrees of relationship among subjects are taken into account in the computation of the variance of the score statistic (see Appendix 1.3), insuring the tests have the correct Type I error rate, as shown in our simulations.

Setting $\underset{\sim}{\beta} = 0$, we obtain:

$$\begin{aligned} \left.\frac{\partial \log P}{\partial \beta_h}\right|_{\underset{\sim}{\beta}=0} &= \sum_{i=1}^{n_1} X_{1i}^{(h)} + \ldots + \sum_{i \in \mathcal{S}_{K-1}} X_{(K-1)i}^{(h)} - \frac{\sum_{\mathcal{S}} \left(\sum_{i \in \mathcal{S}_1} X_{1i}^{(h)} + \ldots + \sum_{i \in \mathcal{S}_{K-1}} X_{(K-1)i}^{(h)} \right)}{\sum_{\mathcal{S}} 1} \\ &= \sum_{i=1}^{n_1} X_{1i}^{(h)} - \frac{\sum_{\mathcal{S}} \sum_{i \in \mathcal{S}_1} X_{1i}^{(h)}}{\binom{n}{n_1,\ldots,n_K}} + \ldots + \sum_{i \in S_{K-1}} X_{(K-1)i}^{(h)} - \frac{\sum_{\mathcal{S}} \sum_{i \in \mathcal{S}_{K-1}} X_{(K-1)i}^{(h)}}{\binom{n}{n_1,\ldots,n_K}} \end{aligned}$$

Each pair of terms above equals a term in equation 3, as shown below:

$$
\begin{aligned}
&\sum_{i\in E_a} X_{ai}^{(h)} - \frac{\sum_{\mathcal{S}}\sum_{i\in\mathcal{S}_a} X_{ai}^{(h)}}{\binom{n}{n_1,\ldots,n_K}} \\
&= \sum_{i\in E_a} X_{ai}^{(h)} - \frac{\sum_{\mathcal{S}_a}\sum_{\mathcal{S}\setminus\mathcal{S}_a}\sum_{i\in\mathcal{S}_a} X_{ai}^{(h)}}{\binom{n}{n_1,\ldots,n_K}} \\
&= \sum_{i\in E_a} X_{ai}^{(h)} - \frac{\sum_{\mathcal{S}_a}\binom{n-n_a}{n_1,\ldots,n_{a-1},n_{a+1},\ldots,n_K}\sum_{i\in\mathcal{S}_a} X_{ai}^{(h)}}{\binom{n}{n_1,\ldots,n_K}} \\
&= \sum_{i\in E_a} X_{ai}^{(h)} - \frac{\sum_{\mathcal{S}_a}\sum_{i\in\mathcal{S}_a} X_{ai}^{(h)}}{\binom{n}{n_a}} \\
&= \frac{1}{n-n_a}\sum_{i\in E_a}\sum_{j\in E_a^c}(X_{ai}^{(h)} - X_{aj}^{(h)}) - \frac{1}{n-n_a}\frac{\sum_{\mathcal{S}_a}\sum_{i\in\mathcal{S}_a}\sum_{j\in E_a^c}(X_{ai}^{(h)} - X_{aj}^{(h)})}{\binom{n}{n_a}} \\
&= \frac{1}{n-n_a}\sum_{i\in E_a}\sum_{j\in E_a^c}(X_{ai}^{(h)} - X_{aj}^{(h)}) - \frac{1}{n-n_a}\frac{\binom{n}{n_a}\frac{n_a}{n}\sum_{i\in E_a}\sum_{j\in E_a^c}(X_{ai}^{(h)} - X_{aj}^{(h)})}{\binom{n}{n_a}} \\
&= \frac{1}{n}\sum_{i\in E_a}\sum_{j\in E_a^c}(X_{ai}^{(h)} - X_{aj}^{(h)})
\end{aligned}
$$

where $E_a = \{n_1 + \cdots + n_{a-1} + 1, \ldots, n_1 + \cdots + n_a\}$ for $a > 1$ and $E_1 = \{1 \ldots n_1\}$. The last three steps correspond in our notation to the derivation in page 374 of Appendix A of **Chen et al.** (2009).

1.2 DERIVATION OF THE PAIRWISE WEIGHTS AS A FUNCTION OF THE FREE PREDICTORS UNDER THE NULL HYPOTHESIS

We adopt the pairwise approach of **Liang and Stewart** (1987), which considers the conditional probability of all pairs of subjects i and j from two outcome categories a and b in the same family:

$$
\begin{aligned}
P_{ij}(a,b) &= P\left[Y_i = a, Y_j = b | Y_i, Y_j \in \{a,b\}\right] \\
&= \frac{\exp\left\{X_{ai}\underset{\sim}{\beta}_a + X_i^{(c)}\underset{\sim}{\alpha}_a + X_{bj}\underset{\sim}{\beta}_b + X_j^{(c)}\underset{\sim}{\alpha}_b\right\}}{\exp\left\{X_{ai}\underset{\sim}{\beta}_a + X_i^{(c)}\underset{\sim}{\alpha}_a + X_{bj}\underset{\sim}{\beta}_b + X_j^{(c)}\underset{\sim}{\alpha}_b\right\} + \exp\left\{X_{bi}\underset{\sim}{\beta}_b + X_i^{(c)}\underset{\sim}{\alpha}_b + X_{aj}\underset{\sim}{\beta}_a + X_j^{(c)}\underset{\sim}{\alpha}_a\right\}}
\end{aligned}
$$

where $\underset{\sim}{\alpha}_K = 0$.

The score for $\underset{\sim}{\beta}_a$ derived from the sum over $P_{ij}(a,b)$ for all pairs of subjects in outcome categories a and b is

$$S_a = \frac{\partial \sum_{i \in E_a} \sum_{j \in E_b} \log P_{ij}(a,b)}{\partial \underset{\sim}{\beta}_a}$$

$$= \sum_{i \in E_a} \sum_{j \in E_b} \left[X_{ai} - \frac{\begin{array}{c} X_{ai} \exp\left\{X_{ai}\underset{\sim}{\beta}_a + X_i^{(c)}\underset{\sim}{\alpha}_a + X_{bj}\underset{\sim}{\beta}_b + X_j^{(c)}\underset{\sim}{\alpha}_b\right\} \\ +X_{aj} \exp\left\{X_{bi}\underset{\sim}{\beta}_b + X_i^{(c)}\underset{\sim}{\alpha}_b + X_{aj}\underset{\sim}{\beta}_a + X_j^{(c)}\underset{\sim}{\alpha}_a\right\} \end{array}}{\begin{array}{c} \exp\left\{X_{ai}\underset{\sim}{\beta}_a + X_i^{(c)}\underset{\sim}{\alpha}_a + X_{bj}\underset{\sim}{\beta}_b + X_j^{(c)}\underset{\sim}{\alpha}_b\right\} \\ + \exp\left\{X_{bi}\underset{\sim}{\beta}_b + X_i^{(c)}\underset{\sim}{\alpha}_b + X_{aj}\underset{\sim}{\beta}_a + X_j^{(c)}\underset{\sim}{\alpha}_a\right\} \end{array}} \right]$$

where $E_a = \{n_1 + \cdots + n_{a-1} + 1, \ldots, n_1 + \cdots + n_a\}$ for $a > 1$, $E_1 = \{1 \ldots n_1\}$ and E_b is defined similarly.

Setting $\underset{\sim}{\beta} = 0$, we obtain

$$S_a|_{\underset{\sim}{\beta}=0} = \sum_{i \in E_a} \sum_{j \in E_b} \left[X_{ai} - \frac{X_{ai} \exp\left\{X_i^{(c)}\underset{\sim}{\alpha}_a + X_j^{(c)}\underset{\sim}{\alpha}_b\right\} + X_{aj} \exp\left\{X_i^{(c)}\underset{\sim}{\alpha}_b + X_j^{(c)}\underset{\sim}{\alpha}_a\right\}}{\exp\left\{X_i^{(c)}\underset{\sim}{\alpha}_a + X_j^{(c)}\underset{\sim}{\alpha}_b\right\} + \exp\left\{X_i^{(c)}\underset{\sim}{\alpha}_b + X_j^{(c)}\underset{\sim}{\alpha}_a\right\}} \right]$$

$$= \sum_{i \in E_a} \sum_{j \in E_b} \frac{(X_{ai} - X_{aj}) \exp\left\{X_i^{(c)}\underset{\sim}{\alpha}_b + X_j^{(c)}\underset{\sim}{\alpha}_a\right\}}{\exp\left\{X_i^{(c)}\underset{\sim}{\alpha}_a + X_j^{(c)}\underset{\sim}{\alpha}_b\right\} + \exp\left\{X_i^{(c)}\underset{\sim}{\alpha}_b + X_j^{(c)}\underset{\sim}{\alpha}_a\right\}}$$

$$= \sum_{i \in E_a} \sum_{j \in E_b} \frac{(X_{ai} - X_{aj})}{1 + \exp\left\{(X_i^{(c)} - X_j^{(c)})(\underset{\sim}{\alpha}_a - \underset{\sim}{\alpha}_b)\right\}}$$

The denominator is proportional to the weight function 6, in the score for the coefficients pertaining to any outcome category a. In practice the coefficients $\underset{\sim}{\alpha}$ are estimated. The additional variability in the score statistic from estimating the $\underset{\sim}{\alpha}$ is neglected here.

An alternative to this pairwise approach would be to use the score obtained from the conditional probabilities of as many subjects as the number of outcome categories observed in each family (between 2 and K) in a fully polytomous approach. While scores obtained from such probabilities would be more efficient to estimate $\underset{\sim}{\beta}$, this is not the purpose here. The $\underset{\sim}{\alpha}$ are not estimated from these scores, they are estimated separately using a polytomous approach. Expressions for the weights derived from the polytomous approach are much more complicated, and are unlikely to differ much from the weights from the pairwise approach with the same $\underset{\sim}{\alpha}$ estimates. It is noteworthy that in families where only two outcome categories are observed the polytomous and pairwise approaches coincide.

1.3 VARIANCE OF THE SCORE STATISTIC FOR A POLYTOMOUS OUTCOME WITH K LEVELS

Expressions for the variance are derived using the locus specific IBD sharing π_{hij} between relatives i and j, estimated from multilocus marker data at each of the two unlinked loci. Using estimated IBD sharing is appropriate for a test of association in the presence of linkage. For a test of

association and linkage, or when IBD is unknown, π_{hij} can be substituted by twice the kinship coefficients ϕ_{ij}, which is the constant at all loci. We also assume that all subjects are outbred.

The variance of the hth element of the score vector is given by the expression:

$$\begin{aligned} Var[S^{(h)}] &= Var\left[\sum_{i=1}^{n_1}\sum_{j=n_1+1}^{n} C_{ij}(X_{1i}^{(h)} - X_{1j}^{(h)}) + \sum_{i\in E_2}\sum_{j\in E_2^c} C_{ij}(X_{2i}^{(h)} - X_{2j}^{(h)}) + \ldots\right] \\ &= \sum_{i=1}^{n_1}\sum_{j=n_1+1}^{n}\sum_{k=1}^{n_1}\sum_{l=n_1+1}^{n} C_{ij}C_{kl}Cov[X_{1i}^{(h)} - X_{1j}^{(h)}, X_{1k}^{(h)} - X_{1l}^{(h)}] \\ &\quad + \sum_{i\in E_2}\sum_{j\in E_2^c}\sum_{k\in E_2}\sum_{l\in E_2^c} C_{ij}C_{kl}Cov[X_{2i}^{(h)} - X_{2j}^{(h)}, X_{2k}^{(h)} - X_{2l}^{(h)}] + \ldots \\ &\quad + \sum_{i=1}^{n_1}\sum_{j=n_1+1}^{n}\sum_{k\in E_2}\sum_{l\in E_2^c} C_{ij}C_{kl}Cov[X_{1i}^{(h)} - X_{1j}^{(h)}, X_{2k}^{(h)} - X_{2l}^{(h)}] + \ldots \end{aligned}$$

where the weights C_{ij} can be defined as in equations 6 or 7 and E_a is the set of indices corresponding to the subjects in level a as in Appendix 1.1. For instance, $E_2 = \{n_1 + 1, \ldots, n_1 + n_2\}$.

Similarly, the covariance between elements g and h of the score vector is given by:

$$\begin{aligned} Cov[S^{(h)}, S^{(g)}] = Cov &\left[\sum_{i=1}^{n_1}\sum_{j=n_1+1}^{n} C_{ij}(X_{1i}^{(h)} - X_{1j}^{(h)}) + \sum_{i\in E_2}\sum_{j\in E_2^c} C_{ij}(X_{2i}^{(h)} - X_{2j}^{(h)}) + \ldots,\right. \\ &\left.\sum_{i=1}^{n_1}\sum_{j=n_1+1}^{n} C_{ij}(X_{1i}^{(g)} - X_{1j}^{(g)}) + \sum_{i\in E_2}\sum_{j\in E_2^c} C_{ij}(X_{2i}^{(g)} - X_{2j}^{(g)}) + \ldots\right] \end{aligned}$$

The covariances $Cov[X_{ai}^{(h)} - X_{aj}^{(h)}, X_{ak}^{(h)} - X_{al}^{(h)}]$ among terms of the same logistic function between level a and K are given in equation 9.

The covariances $Cov[X_{ai}^{(h)} - X_{aj}^{(h)}, X_{bk}^{(g)} - X_{bl}^{(g)}], a \neq b$ are 0 when $X_a^{(h)}$ is not defined because β_h is not involved in the logistic function between levels a and K or $X_b^{(g)}$ is not defined because β_g is not involved in the logistic function between levels b and K. When β_h for level a multiplies the same term X_p as β_g for level b then $X_a^{(h)} = X_b^{(g)} = X_p$ (or X_p is the non-empty intersection of the two terms, *e.g.* X_1 and $X_1(1 - X_2) = X_1 - X_1X_2$ have intersection X_1). The covariance is then

$$\begin{aligned} Cov[X_{ai}^{(h)} - X_{aj}^{(h)}, X_{bk}^{(g)} - X_{bl}^{(g)}] &= Cov[X_{pi} - X_{pj}, X_{pk} - X_{pl}] \qquad (1) \\ &= Cov[X_{pi}, X_{pk}] + Cov[X_{pj}, X_{pl}] - Cov[X_{pi}, X_{pl}] - Cov[X_{pj}, X_{pk}] \end{aligned}$$

The covariances $Cov[X_{ai}^{(h)} - X_{aj}^{(h)}, X_{bk}^{(h)} - X_{bl}^{(h)}], a \neq b$ are 0 when either $X_a^{(h)}$ or $X_b^{(h)}$ is not defined because β_h is not involved in either the logistic function between levels a and K or the one between levels b and K. When $X_a^{(h)} = X_b^{(h)} = X_p$, *i.e.* the coefficient β_h is common to the term X_p from the logistic functions for level a and for level b, then equation 1 above applies.

1.4 VARIANCE AND COVARIANCE TERMS

The expressions for $Var[X_{pi}]$ and $Cov[X_{pi}, X_{pj}]$ depend on the nature of the term X_p. When X_p corresponds to the main effect of a locus, say X_1, then

$$Var[X_{1i}] = \sigma_1^2$$

and the covariance between two related subjects is

$$Cov[X_{1i}, X_{1j}] = \pi_{1ij}\sigma_1^2$$

Now consider the computation of $Var[X_{pi}]$ and $Cov[X_{pi}, X_{pj}]$ when the term X_p is a product term of the allele counts of locus 1 and 2. We expand the interaction term as a product of the allelic counts X_1 and X_2. We therefore reformulate $X_{pi} \equiv X_{1i}X_{2i}$. We denote the variance

$$Var[X_{1i}X_{2i}] = \sigma_{12}^2$$

We need here the assumption that locus 1 and 2 are unlinked to write the covariance between two related subjects as

$$Cov[X_{1i}X_{2i}, X_{1j}X_{2j}] = \pi_{1ij}\pi_{2ij}\sigma_{12}^2$$

1.5 ESTIMATION OF σ^2

Following **Chen et al.** (2009), we define estimates of σ_l^2 for each level a as:

$$\hat{\sigma}_l^{2(a)} = \frac{\sum_{i \in E_a} \sum_{j \in E_a^c} (X_{li} - X_{lj})^2}{\sum_{i \in E_a} \sum_{j \in E_a^c} 2(\phi_{ii} + \phi_{jj} - \pi_{hij})}$$

where ϕ_{ii} is the inbreeding coefficient of subject i, with $\phi_{ii} = \frac{1}{2}$ when i is outbred.

We extend this moment estimator to product terms as follows:

$$\hat{\sigma}_{l_1,l_2}^{2(a)} = \frac{\sum_{i \in E_a} \sum_{j \in E_a^c} (X_{l_1 i}X_{l_2 i} - X_{l_1 j}X_{l_2 j})^2}{\sum_{i \in E_a} \sum_{j \in E_a^c} 2(\phi_{ii} + \phi_{jj} - \pi_{h_1 ij}\pi_{h_2 ij})}$$

For variance terms involving a single logistic function, *e.g.* terms from $Var[S^{(h)}]$ for β_h belonging only to the logistic function between levels a and K, we estimate σ^2 by $\hat{\sigma}^{2(a)}$. For variance terms involving two logistic functions *e.g.* terms from $Cov[S^{(h)}, S^{(g)}]$ for a coefficient β_h belonging to logistic function between levels a and K and β_g belonging to logistic function between levels b and K, we have two relevant estimates, $\hat{\sigma}^{2(a)}$ and $\hat{\sigma}^{2(b)}$. We then take the mean of these two estimates $\hat{\sigma}^{2(ab)} = \frac{1}{2}(\hat{\sigma}^{2(a)} + \hat{\sigma}^{2(b)})$.

1.6 COMPUTATION OF THE IBD TERMS

The computation of the quadruple summation in the expressions for the variance such as equation 9 requires a quadruple loop over the four indices when pairwise weights C_{ij} are used. Without weights, the following simplification can be used. We drop the locus subscript to simplify the notation.

$$\sum_{i\in E_a}\sum_{j\in E_a^c}\sum_{k\in E_b}\sum_{l\in E_b^c}(\pi_{ik}+\pi_{jl}-\pi_{il}-\pi_{jk}) = (n-n_a)(n-n_b)\sum_{i\in E_a}\sum_{k\in E_b}\pi_{ik}+n_a n_b\sum_{j\in E_a^c}\sum_{l\in E_b^c}\pi_{jl} -n_b(n-n_a)\sum_{i\in E_a}\sum_{l\in E_b^c}\pi_{il}-n_a(n-n_b)\sum_{j\in E_a^c}\sum_{k\in E_b}\pi_{jk}$$

In particular, when the two levels (categories) are the same, then $E_a = E_b$ and $n_a = n_b$. Using the symmetry of the π_{ij}'s, we have

$$\sum_{i\in E_a}\sum_{j\in E_a^c}\sum_{k\in E_b}\sum_{l\in E_b^c}(\pi_{ik}+\pi_{jl}-\pi_{il}-\pi_{jk}) = (n-n_a)^2\sum_{i,k\in E_a}\pi_{ik}+n_a^2\sum_{j,l\in E_a^c}\pi_{jl} -2n_a(n-n_a)\sum_{i\in E_a}\sum_{l\in E_a^c}\pi_{il}$$

REFERENCES

Chen, W. M., Manichaikul, A., and Rich, S. S. (2009), A generalized family-based association test for dichotomous traits, *Am J Hum Genet*, 85, 3, 364–76

Liang, K.-Y. and Stewart, W. F. (1987), Polychotomous logistic regression methods for matched case-control studies with multiple case or conrol groups, *AJE*, 125, 4, 720–30
